# Two Variants in the *NOTCH4* and *HLA-C* Genes Contribute to Familial Clustering of Psoriasis

**DOI:** 10.1155/2020/6907378

**Published:** 2020-10-19

**Authors:** Minglong Cai, He Huang, Zhulin Hu, Tao Yuan, Weiran Li, Yaoguang Liu, Lijun Zheng, Yan Zhang, Yujun Sheng, Xuejun Zhang

**Affiliations:** ^1^Department of Dermatology, The First Affiliated Hospital of Anhui Medical University, Hefei 230032, China; ^2^Institute of Dermatology, Anhui Medical University, Hefei 230032, China

## Abstract

Psoriasis is a multifactorial immune-mediated skin disease with a strong genetic background. Previous studies reported that psoriasis with a family history (PFH) and sporadic psoriasis (SP) have a distinct manifestation and genetic predisposition. However, the genetic heterogeneity of PFH and SP in the major histocompatibility complex (MHC) region has not been fully elucidated. To explore genetic variants in the MHC region that drive family aggregation of psoriasis, we included a total of 8,127 psoriasis cases and 9,906 healthy controls from Han Chinese and divided psoriasis into two subtypes, PFH (*n* = 1,538) and SP (*n* = 5,262). Then, we calculated the heritability of PFH and SP and performed a large-scale stratified association analysis. We confirmed that variants in the MHC region collectively explained a higher heritability of PFH (16.8%) than SP (13.3%). Further stratified association analysis illustrated that *HLA-C*∗*06:02* and *NOTCH4:G511S* contribute to the family aggregation of psoriasis, and *BTNL2:R281K* specifically confers risk for SP. *HLA-C*∗*06:02* and *NOTCH4:G511S* could partially explain why patients with PFH have a stronger genetic predisposition, more complex phenotypes, and more frequent other autoimmune diseases. The identification of the SP-specific variant *BTNL2:R281K* revealed that the genetic architecture of SP is not just a subset of PFH.

## 1. Introduction

Psoriasis is a chronic and multifactorial immune-mediated skin disease characterized by epidermal hyperplasia. It affects 0.09%–11.4% of worldwide population [1, 2]. Psoriasis has a strong genetic predisposition. Evidence shows monozygotic twins have higher concordance rates (0.2–0.73) than dizygotic twins (0.09–0.2) [3–8]. Moreover, 23.1%–31.9% of psoriasis patients have a family history [9, 10]. To date, linkage analysis and genome-wide association studies (GWAS) have identified more than 80 disease-associated loci, with the most prominent risk loci observed in the human leukocyte antigen (HLA) region [11–14].

Although patients with psoriasis tend to cluster in families, most of them are sporadic. Evidence has shown psoriasis with a family history (PFH) is different from sporadic psoriasis (SP) in terms of both genetic background and phenotype. Genes outside of the major histocompatibility complex (MHC) region such as *ERAP1* and *NFKB1* were found to be associated with the risk of familial aggregation of psoriasis [15, 16]. A clinical investigation reported that PFH patients have an earlier onset of psoriasis and more frequently nail disease, enthesitis, and other autoimmune diseases [10, 17]. It seems that PFH has a stronger genetic background and more complicated phenotypes than SP.

However, the effects of the genetic heterogeneity of the HLA alleles on PFH and SP had been largely underexplored in spite of the strong associations between HLA alleles and psoriasis. One of the greatest challenges is that HLA genes are highly polymorphic and can be in strong linkage disequilibrium, which has complicated the determination of the independent risk signals. In this study, we devised an analytical approach to identify the heterogeneous effects of HLA alleles on PFH and SP by deep sequencing of the MHC region. We first calculated the heritability of PFH and SP and confirmed that variants in the MHC region collectively explained a higher heritability of PFH than SP. Then, we considered two hypothetical models of the genetic structure in PFH versus SP: (1) We considered that shared variants might be associated with both PFH and SP; such variants would confer more risk of PFH than of SP. (2) We considered that specific variants might specifically confer risk for PFH; such variants would not be associated with SP. Based on two hypothetical models, we conducted a step-wise association analysis of HLA with PFH and SP to identify those shared and specific variants for psoriasis subtypes. Finally, we performed a case-case analysis (PFH vs. SP) to determine whether those shared and specific variants contribute to the family aggregation of psoriasis.

## 2. Materials and Methods

### 2.1. Participants

We included a total of 8,127 patients with psoriasis (patients with psoriasis arthritis were not included) and 9,906 geographically matched healthy controls of Chinese ancestry, as previously described [18]. All cases were diagnosed by at least two dermatologists, and all controls were healthy individuals without psoriasis, any autoimmune diseases, or a family history of genetic diseases (including first-, second- and third-degree relatives). Clinical information for all individuals was gathered using a previously developed structured questionnaire. All participants signed informed consent. The study was approved by the institutional ethical committee of the First Affiliated Hospital of Anhui Medical University (No.20131349) and was conducted in accordance with the Declaration of Helsinki principles.

### 2.2. Sequencing and Quality Control

The samples enrolled for our analysis underwent targeted sequencing and stringent quality control as described in our previous study [18]. The raw sequencing data from samples have been deposited in the Sequence Read Archive (SRA) under the accession number SRA205317. Furthermore, we removed variants with a call rate < 99%, MAF < 0.5%, or Hardy–Weinberg equilibrium of *P* < 10^−4^. A total of 26,775 variants remained for the following analysis.

### 2.3. Phenotype Classification

We classified psoriasis (*n* = 8,127) into two subtypes, PFH (*n* = 1,722) and SP (*n* = 6,405). To rule out the influence of the confounder of the age of onset, we removed patients with age of onset after 40 years, retaining 1,538 PFH and 5,262 SP for the following analysis.

### 2.4. Statistical Analyses

The association of age of onset with a family history of psoriasis was evaluated by the Pearson chi-square test and Student's *t* test. The association analysis of HLA with psoriasis subtypes was performed using logistic regression, assuming an additive effect of the allele dosages in the log-odds scale. We included sex and region as covariates to adjust for population stratification. Association analysis was conducted as follows: (1) association analysis of variants with PFH and (2) association analysis of variants with SP. For conditional association analysis, we consecutively included the detected variants as covariates to search for additional variants with independent association. This was repeated in a forward stepwise approach until no marker satisfied the significance threshold (Bonferroni correction: 0.05/26,775 = 1.87 × 10^−6^). For those variants that were independently associated with PFH or SP, we further conducted a case-case association analysis by using logistic regression after adjusting for sex and region. For epistasis analysis, we defined a logistic regression model that simultaneously included interaction terms between significant variants. Association analysis and conditional association analysis were conducted by PLINK (v.1.9) [19]. Plots of HLA associations were drawn by R (v.3.6.1).

### 2.5. Annotation of Variants

For variants with amino acid changes, we used SIFT [20], PolyPhen-2 [21], CADD [22], and DANN [23] to measure whether an amino acid is deleterious based on changes in the structure/function of proteins, allelic diversity, pathogenicity, and conservation. The criteria used are as follows: SIFT: deleterious (sift_score ≤ 0.05), tolerated (sift_score > 0.05); PolyPhen-2: probably damaging (pp2_hdiv ≥ 0.957), possibly damaging (0.453 ≤ pp2_hdiv ≤ 0.956); benign (pp2_hdiv ≤ 0.452); CADD: top 10% most deleterious (scaled C − scores ≥ 10); top 1% most deleterious (scaled C − scores ≥ 20); DANN: top 10% most deleterious (DANN_score ≥ 0.9); top 1% most deleterious (DANN_socre ≥ 0.99).

## 3. Results

### 3.1. Sample Selection and Phenotype Classification

A total of 8,127 psoriasis patients were included. Clinically, psoriasis can be classified into type 1 psoriasis (defined as age of onset before 40) and type 2 psoriasis (defined as age of onset after 40) [24]. It is well known that genetic factors are more prevalent in type 1 psoriasis, and type 1 psoriasis is more likely to be familial [24]. In our study, we observed that type 1 psoriasis was strongly associated with familial aggregation (OR = 1.816, *P* = 9.58 × 10^−13^; Table 1). In order to rule out the influence of age of onset, we excluded patients with age of onset after 40, retaining 1,538 PFH (mean age of onset: 21.79 ± 9.19) and 5,262 SP (mean age of onset: 22.26 ± 6.97) cases for the following analysis. There was no substantial correlation between age of onset and family history (*P* = 0.061) after removing patients with type 2 psoriasis.

### 3.2. Variants Identification

Our target sequencing of the entire 5-Mb MHC region, from upstream of *HLA-A* to downstream of *HLA-DPB1*, detected a total of 36,300 indels and 224,872 SNPs [18]. Next, we generated accurate genotyping results (i.e., HLA alleles, amino acid polymorphisms, etc.) for genes including the HLA classic genes (i.e., *HLA-A*, *HLA-B*, *HLA-C*, etc.) and the non-HLA genes (i.e., *NOTCH4*, *TAP2*, *MICA*, etc.). After stringent quality control as previously described [18], we further removed variants with a call rate < 99%, minor allele frequency (MAF) < 0.5%, or Hardy–Weinberg equilibrium of *P* < 10^−4^. A total of 26,775 variants, including HLA alleles, amino acid polymorphisms, SNPs, and short indels remained for the following analysis, with most of them (63.6%) being common variants (MAF > 5%).

### 3.3. Heritability of PFH and SP

To determine whether the genetic background of PFH is stronger than that of SP, GCTA (v1.93.1) [25] was used to calculate the heritability of PFH and SP under the assumption of a disease prevalence of 0.47%. We observed that variants in the MHC region collectively explained a higher heritability of PFH (*h*^2^ = 16.8%, standard error = 0.0158) than SP (*h*^2^ = 13.3%, standard error = 0.0095). This confirmed that PFH has a stronger genetic predisposition than SP in terms of the MHC region.

### 3.4. Associations of HLA with PFH

**Figure 1:**
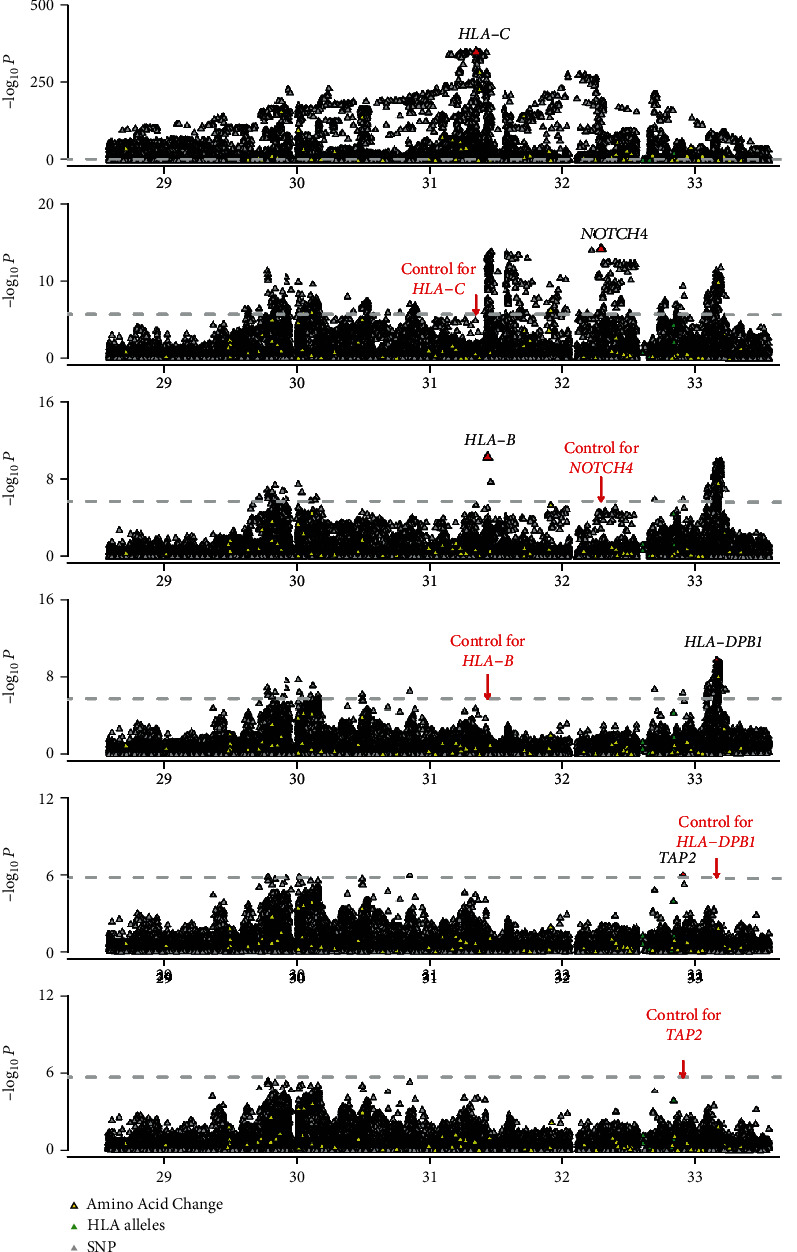
A Manhattan plot of the stepwise conditional association for psoriasis with a family history (PFH) in the major histocompatibility complex (MHC) region. Stepwise analysis of *HLA-C*, *NOTCH4*, *HLA-B*, *HLA-DPB1*, and *TAP2* in PFH vs. control. For each plot, the horizontal axis shows the genomic position, and the vertical axis shows negative log_10_-transformed *P* values for association. The dashed horizontal line corresponds to the significance threshold of *P* = 1.87 × 10^−6^. The red triangles represent the top associated variants.

We performed a stepwise association analysis of variants with PFH susceptibility (1,538 PFH vs. 9,906 control). The most significantly associated variant mapped to the classical *HLA-C*∗*06:02* allele (OR = 12.92, *P* = 1.00 × 10^−352^; Figure 1, Table 2), followed by *HLA-C*∗*07:04* (OR = 3.19, *P* = 6.63 × 10^−10^). After conditioned on *HLA-C*∗*06:02* and *HLA-C*∗*07:04*, there was no variant in *HLA-C* that reached the predefined threshold (*P* = 1.87 × 10^−6^). To look for additional independent variants, we conditioned on the *HLA-C* region and observed a novel significant risk variant for *NOTCH4:G511S* (OR = 2.15, *P* = 7.22 × 10^−15^). When we conditioned on *HLA-C* and *NOTCH4*, we observed an independent association at *HLA-B* amino acid position 67 (*B-Y67C*, OR = 1.80, *P* = 5.45 × 10^−11^), which was previously reported to be associated with psoriasis in both European and Han Chinese populations [18, 26]. After conditioning on *HLA-C*, *NOTCH4*, and *HLA-B*, we discovered an independent association for *HLA-DPB1* amino acid position 35 (*DPB1-F35L*, OR = 1.38, *P* = 1.86 × 10^−10^). *DPB1-F35L* was in strong linkage disequilibrium with a previously reported psoriasis-associated allele, *HLA-DPB1*∗*05:01* (*r*^2^ = 0.76) [18]. Further stepwise analysis identified another significant allele for *TAP2:01:03* (OR = 0.41, *P* = 1.02 × 10^−06^). When we conditioned on all the above genes, no additional variant remained significantly associated with PFH susceptibility.

### 3.5. Associations of HLA with SP

**Figure 2:**
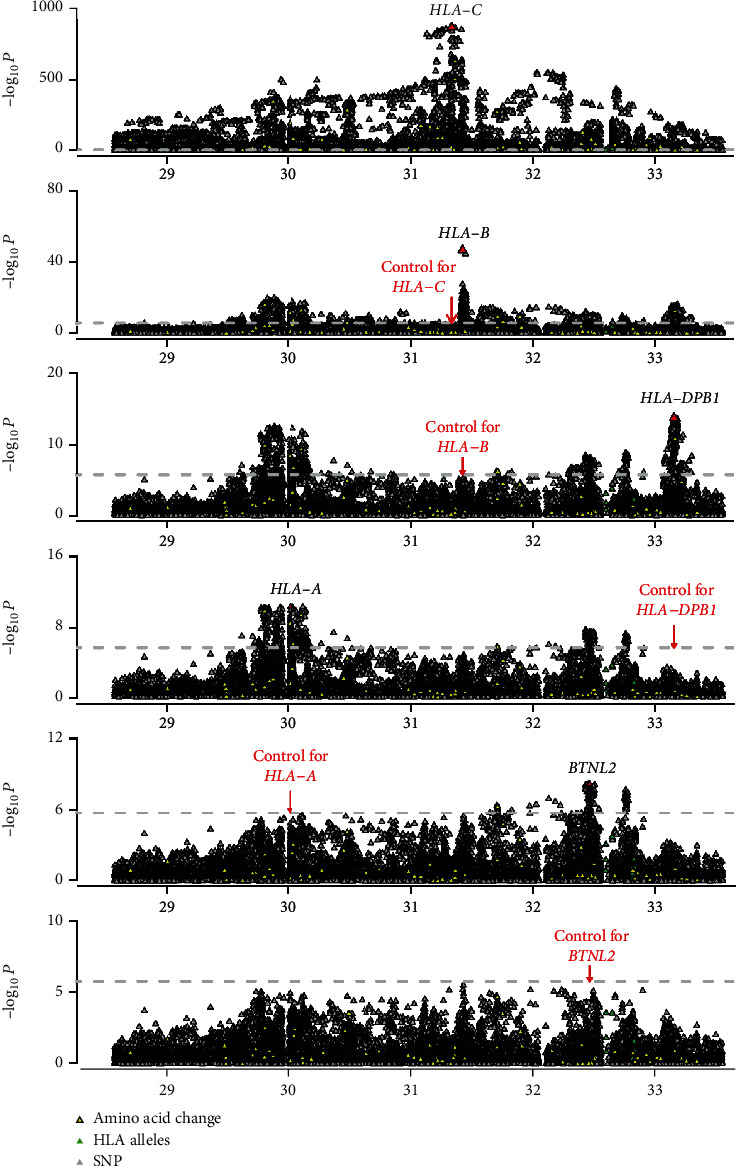
A Manhattan plot of the stepwise conditional association for SP in the MHC region. Stepwise analysis of *HLA-C*, *HLA-B*, *HLA-DPB1*, *HLA-A*, and *BTNL2* in SP vs. control. For each plot, the horizontal axis shows the genomic position and the vertical axis shows the negative log_10_-transformed *P* values for the association. The dashed horizontal line corresponds to the significance threshold of *P* = 1.87 × 10^−6^. The red triangles represent the top associated variants.

As expected, in the fine-mapping analysis of SP (5,262 SP cases vs. 9,906 controls), we observed *HLA-C*∗*06:02* (OR = 15.16, *P* = 1.00 × 10^−863^, Figure 2, Table 3) as the most significantly SP-associated allele, followed by *HLA-C*∗*07:04* (OR = 3.89, *P* = 5.35 × 10^−27^). After the condition on *HLA-C*∗*06:02* and *HLA-C*∗*07:04*, we found an independent association for *HLA-C*∗*01:02* (OR = 1.29, *P* = 8.35 × 10^−8^), which was recently reported to be associated with psoriasis in southern China [27]. We then investigated additional risk variants independent of *HLA-C*. When we included *HLA-C* as covariant, we observed significant independent associations at *HLA-B* amino acids 67 (*B-Y67C*, OR = 2.26, *P* = 2.30 × 10^−47^), 116 (*B-Y116S*, OR = 1.38, *P* = 7.82 × 10^−20^), and 70 (*B-Q70K*, OR = 0.46, *P* = 7.71 × 10^−10^). When we conditioned on *HLA-C* and *HLA-B*, we discovered a significant allele for *HLA-DPB1*∗*05:01* (OR = 1.29, *P* = 5.51 × 10^−15^). Then, we conditioned on *HLA-C*, *HLA-B*, and *HLA-DPB1*. We observed that *HLA-A* amino acid 95 (*A-I95V*, OR = 1.27, *P* = 8.99 × 10^−11^) exceeded the significance threshold. Further stepwise conditional analysis found that *BTNL2:R281K* (OR = 1.46, *P* = 7.18 × 10^−9^) was an independent risk variant. After conditioning on all the above genes, we observed no other significant associations.

### 3.6. Case-Case Association Analysis

Fine-mapping analysis of PFH and SP has identified a total of 11 independent susceptibility variants. To further determine if these 11 variants contribute to the family aggregation of psoriasis, we conducted a case-case analysis (PFH vs. SP), using a *P* value of 4.54 × 10^−3^ (Bonferroni correction for 11 variants, 0.05/11) as the cutoff for statistical significance. We found that *HLA-C*∗*06:02* (OR = 1.33, *P* = 2.63 × 10^−5^) and *NOTCH4:G511S* (OR = 1.24, *P* = 4.26 × 10^−3^) confer risk for PFH, while *BTNL2:R281K* (OR = 0.74, *P* = 2.05 × 10^−3^) confers risk for SP (Table 4).

### 3.7. Annotation of *NOTCH4:G511S* and *BTNL2:R281K*

For variants *NOTCH4:G511S* and *BTNL2:R281K*, we used four annotation programs (see Materials and Methods) to measure the deleteriousness of variants. SIFT predicted that *NOTCH4:G511S* (sift_score = 0.04) and *BTNL2:R281K* (sift_score = 0.04) are deleterious. PolyPhen-2 predicted that *BTNL2:R281K* is probably damaging (pp2_hdiv = 0.999), and *NOTCH4:G511S* is possible damaging (pp2_hdiv = 0.847), respectively. Both CADD and DANN predicted that *BTNL2:R281K* (scaled C − score = 29.7 and DANN_score = 0.997) and *NOTCH4:G511S* (scaled C − score = 23.3 and DANN_score = 0.997) are the top 1% most deleterious variants.

### 3.8. No Epistasis between *HLA-C*∗*06:02* and *NOTCH4:G511S*

Our stratified analysis suggested that *NOTCH4:G511S* and *HLA-C*∗*06:02* contribute to the familial clustering of psoriasis. To further investigate whether there is an interaction between *NOTCH4:G511S* and *HLA-C*∗*06:02*, we performed an epistasis analysis by including interaction terms in the logistic regression model. In the case-case analysis, we included an interaction term between *NOTCH4:G511S* and *HLA-C*∗*06:02* in our logistic regression model, and we observed no evidence of interaction between *NOTCH4:G511S* and *HLA-C*∗*06:02* (*P* = 0.648). In the PFH versus control analysis, 15 interaction terms between all 6 significant variants were included in the logistic regression model; a *P* value of 0.003 (0.05/15) was considered as statistical significance. We found strong interactions between *HLA-C*∗*06:02* and *TAP2*∗*01:03* (OR = 0.35, *P* = 1.7 × 10^−9^) and *HLA-C*∗*06:02* and *DPB1-F35L* (OR = 1.57, *P* = 1.7 × 10^−6^). But we still observed no evidence of interaction between *HLA-C*∗*06:02* and *NOTCH4:G511S* after Bonferroni correction (OR = 0.55, *P* = 0.007). Our results suggested that *HLA-C*∗*06:02* and *NOTCH4:G511S* may independently contribute to the familial clustering of psoriasis.

## 4. Discussion

In this study, we performed a large-scale stratified analysis to detect HLA and non-HLA variants in the MHC region that drive familial aggregation of psoriasis. In fine-mapping analysis of PFH and SP, we identified a total of 11 independent variants, of which eight variants (*C*∗*06:02*, *C*∗*07:04*, *C*∗*01:02*, *HLA-B* amino acid positions 67, 116, *HLA-A* amino acid position 95, *DPB1*∗*05:01*, and *BTNL2:R281K*) were reported to be associated with psoriasis by previous studies [18, 26–28]. Notably, *HLA-C*∗*06:02*, *HLA-C*∗*07:04*, *HLA-B* amino acid position 67, and *HLA-DPB1*∗*05:01* (strong linkage disequilibrium with *DPB1-F35L*) were shared susceptibility variants between PFH and SP, while other variants (*NOTCH4:G511S* and *TAP2*∗*01:03* for PFH, *C*∗*01:02*, *B-Y116S*, *B-Q70K, A-I95V*, and *BTNL2:R281K* for SP) showed specific associations with one of two psoriasis subtypes. Here, we have successfully decomposed the genetic architecture of PFH and SP into shared components and specific components. In order to further determine whether these shared or specific variants contribute to the family aggregation of psoriasis, we conducted a case-case analysis (PFH vs. SP) and eventually identified two variants (*HLA-C*∗*06:02* and *NOTCH4:G511S*) in the MHC region which confer risk for family aggregation of psoriasis. Moreover, we also identified a variant (*BTNL2:R281K*) that specifically confers risk for SP.

For those shared variants between PFH and SP, only *HLA-C*∗*06:02* was validated to be associated with family aggregation of psoriasis in the case-case analysis (OR = 1.33, *P* = 2.63 × 10^−5^). *HLA-C*∗*06:02* is a well-known psoriasis-risk allele, and it was reported to be strongly associated with early age of onset of psoriasis [29]. This might explain why PFH have an earlier onset of psoriasis. Therefore, *HLA-C*∗*06:02* conforms to our first hypothetical model that the shared variant contributes more risk to PFH than to SP. For those specific variants in PFH or SP, we found that *NOTCH4:G511S* (OR = 1.24, *P* = 4.26 × 10^−3^) contributes to the familial clustering of psoriasis. *NOTCH4:G511S* was proven to be an independent risk variant for PFH because the effect of *NOTCH4:G511S* with respect to PFH vs. control risk remained significant even after we conditioned on *HLA-C*, *HLA-B*, *HLA-DPB1*, *HLA-A*, *TAP2*, and *BTNL2* (*P* = 1.62 × 10^−12^, Table S1A). In contrast, the impact of *NOTCH4:G511S* on SP vs. control risk disappeared after we conditioned on *HLA-C* and *HLA-B* (*P* > 1.87 × 10^−6^). Therefore, *NOTCH4:G511S* conforms to the second hypothetical model that the specific variant only confers risk for PFH and not for SP. *NOTCH4* encodes members of the notch family of proteins that positively regulate notch signaling. The notch signaling pathway is a highly conserved molecular network that has a key role in angiogenesis [30], cellular proliferation and differentiation [31], and apoptosis [32]. Other studies have reported that the notch pathway regulates several critical aspects of epidermal renewal and development, including proliferation, differentiation, and cell-fate of keratinocytes [33–36]. For example, a previous study showed that notch signaling induces differentiation of spinous cells into granulosa cells and, simultaneously, prevents premature differentiation of spinous cells, orchestrating the balance between differentiation and immature programs in suprabasal cells during epidermal development [36]. Moreover, the amino acid substitution glycine > serine at position 511 in *NOTCH4* was predicted to be deleterious by all four annotation tools, suggesting this variant (*NOTCH4:G511S*) may cause aberrations in notch signaling and further lead to aggressive angiogenesis and the disruption of keratinocyte differentiation in psoriasis lesions. In addition, previous studies reported that *NOTCH4* was associated with multiple autoimmune diseases, including Crohn's disease, systemic scleroderma, rheumatoid arthritis, and ulcerative colitis [37–40]. Of note, enthesitis is a key early manifestation of psoriasis arthritis. *NOTCH4* was also reported to be associated with psoriasis arthritis [41], and notch signaling pathways were found to mediate synovial angiogenesis in psoriatic arthritis [42]. These findings suggested that notch signaling pathways were involved in the pathogenesis of PFH and *NOTCH4:G511S* may partially explain why PFH has more frequent enthesitis and other autoimmune diseases. We also conducted an epistasis analysis, finding no evidence of interaction between *NOTCH4:G511S* and *HLA-C*∗*06:02*. Therefore, *NOTCH4:G511S* and *HLA-C*∗*06:02* may independently contribute to the family clustering of psoriasis based on our two hypothetical models.

Besides, we identified a variant (*BTNL2:R281K*) that specifically confers risk for SP. The amino acid substitution arginine > lysine at position 281 in *BTNL2* was predicted to be deleterious by all four annotation tools. Notably, this variant was not associated with either PFH (*P* = 4.29 × 10^−5^) or SP (*P* = 0.19) in the univariate analysis (Table S1A). However, it exhibited a significant association with SP after conditioning on *HLA-C*∗*06:02* (*P* = 6.8 × 10^−7^). Subsequently, we classified SP and control into two subgroups based on the presence of *HLA-C*∗*06:02*, the C∗06:02-positive group and the C∗06:02-negative group. We found that although the raw frequency of *BTNL2:R281K* in SP (0.059) was lower than in controls (0.063), this was reversed in both subgroups (0.086 in SP and 0.070 in controls for the C∗06:02-positive group, and 0.054 in SP and 0.036 in controls for C∗06:02-negative group, Table S1B). Moreover, univariate analysis in these two subgroups showed a consistent effect (*P* = 0.01 for the C∗06:02-negative group, *P* = 1.52 × 10^−5^ for the C∗06:02-positive group). This phenomenon can be explained by Simpson's paradox [43], where two subgroups have the same association, but the overall population shows no association. In contrast, *BTNL2:R281K* did not reach the significance threshold in PFH vs. control after conditioning on other susceptibility variants. The case-case analysis also showed that the frequency of *BTNL2:R281K* was significantly higher in SP than in PFH (*P* = 2.05 × 10^−3^). Therefore, *BTNL2:R281K* is an SP-specific risk variant that does not conform to our hypothetical models, which suggests that the genetic architecture of SP is not just a subset of PFH despite the fact that PFH has a stronger genetic predisposition than SP. We speculate that *BTNL2:R281K* might be associated with certain phenotypes of SP. For example, a recent study found that patients with SP have a higher Body Mass Index (BMI) than those with PFH [44]. *BTNL2* was reported to be associated with the waist–hip ratio [45] and might contribute to higher BMI in SP.

However, there are limitations to our study. We were unable to perform further genotype-phenotype analysis because the included samples lacked clinical information, such as BMI, enthesitis, and other phenotypes of psoriasis. Besides, the functional mechanisms by which these variants contribute to familial aggregation of psoriasis or disease phenotypes remain unclear. Further studies will be required to address these problems.

In conclusion, we have identified two variants that independently contribute to family clustering of psoriasis, and one variant specifically confers risk for SP. We found *NOTCH4:G511S* and *HLA-C*∗*06:02* could partially explain why patients with a family history have an earlier onset of psoriasis and more frequent enthesitis and other autoimmune diseases. Our findings support the hypothetical model that PFH has a stronger genetic background than SP, but also indicate that the genetic architecture of SP is not just a subset of PFH. Three variants identified in our study enable to differentiate PFH from SP and might serve as markers to predict the risk of developing psoriasis for individuals at a young age who have a family history.

## Figures and Tables

**Table 1:** Description of study samples of patients with psoriasis.

Subtypes	Age of onset before removing type 2 psoriasis				Age of onset after removing type 2 psoriasis	
	≤40	>40	OR	*P*	Mean ± SD^1^	*P*
PBH	1,538	184	1.82	9.58 × 10^−13^	21.79 ± 9.19	0.061
SP	5,262	1,143			22.26 ± 6.97	

^1^SD: standard deviation.

**Table 2:** Association of variants with psoriasis with a family history (PFH) in the multivariate regression model.

Variants	Frequency		PFH vs. control	
	Freq_PFH^1^	Freq_Control^2^	OR (95% CI)	*P* value
HLA-C alleles				
*HLA-C*∗*06:02*	0.479	0.110	12.92 (11.41–14.64)	1.00 × 10^−352^
*HLA-C*∗*07:04*	0.015	0.010	3.19 (2.21–4.61)	6.63 × 10^−10^
NOTCH4				
*NOTCH4:G511S*	0.092	0.018	2.15 (1.77–2.61)	7.22 × 10^−15^
HLA-B alleles				
*B-Y67C*	0.082	0.087	1.80 (1.51–2.15)	5.45 × 10^−11^
HLA-DPB1 alleles				
*DPB1-F35L*	0.407	0.437	1.38 (1.25–1.52)	1.86 × 10^−10^
TAP2				
*TAP2*∗*01:03*	0.028	0.025	0.41 (0.29–.59)	1.02 × 10^−6^

^1^Freq_PFH: the allele frequency in patients with PFH. ^2^Freq_Control: the allele frequency in healthy controls.

**Table 3:** Association of variants with sporadic psoriasis (SP) in the multivariate regression model.

Variants	Frequency		SP vs. control	
	Freq_SP^1^	Freq_Control^2^	OR (95% CI)	*P* value
HLA-C alleles				
HLA-C∗06:02	0.444	0.110	15.16 (13.93–16.49)	1.00 × 10^−863^
HLA-C∗07:04	0.0148	0.010	3.89 (3.04–4.99)	5.35 × 10^−27^
HLA-C∗01:02	0.107	0.139	1.29 (1.18–1.42)	8.35 × 10^−08^
HLA-B alleles				
B-Y67C	0.093	0.087	2.26 (2.03–2.53)	2.30 × 10^−47^
B-Y116S	0.316	0.326	1.38 (1.29–1.48)	7.82 × 10^−20^
B-Q70K	0.016	0.023	0.46 (0.36–0.59)	7.71 × 10^−10^
HLA-DPB1 alleles				
DPB1∗05:01	0.362	0.364	1.29 (1.21–1.38)	5.51 × 10^−15^
HLA-A alleles				
A-I95V	0.257	0.299	1.27 (1.18–1.37)	8.89 × 10^−11^
BTNL2				
BTNL2:R281K	0.059	0.063	1.46 (1.29–1.66)	7.18 × 10^−9^

^1^Freq_SP: the allele frequency in patients with SP. ^2^Freq_Control: the allele frequency in healthy controls.

**Table 4:** Association of human leukocyte antigen (HLA) with a family history of psoriasis.

Variants	Frequency		PFH vs. SP	
	Freq_PFH	Freq_SP	OR (95% CI)	*P* value
*HLA-C*∗*06:02^1^*	*0.479*	*0.444*	*1.33 (1.17–1.52)*	2.63 × 10^−5^
HLA-C∗07:04	0.015	0.015	0.92 (0.65–1.29)	0.624
HLA-C∗01:02	0.092	0.107	0.93 (0.80–1.07)	0.295
B-Y67C	0.081	0.092	0.86 (0.74–0.998)	0.047
B-Y116S	0.313	0.316	1.01 (0.92–1.1)	0.920
B-Q70K	0.016	0.016	1.03 (0.74–1.44)	0.855
DPB1∗05:01	0.354	0.362	0.98 (0.89–1.07)	0.591
A-I95V	0.250	0.257	0.95 (0.86–1.05)	0.287
*NOTCH4:G511S^1^*	*0.092*	*0.076*	*1.24 (1.07–1.44)*	4.26 × 10^−3^
*BTNL2:R281K^1^*	*0.044*	*0.059*	*0.74 (0.61–0.89)*	2.05 × 10^−3^
TAP2∗01:03	0.028	0.026	1.04 (0.81–1.35)	0.76

^1^Variants with *P* < 4.54 × 10^−3^ in the significant threshold in case-case analysis and are marked in italics.

## Data Availability

The raw sequencing data from samples have been deposited in the Sequence Read Archive (SRA) under the accession SRA205317 (http://www.ncbi.nlm.nih.gov/sra/).

## Supplementary Materials

Supplementary Materials Table S1A: Detailed stepwise association results of *NOTCH4:G534S* and *BTNL2:R281K* with psoriasis risk. Table S1B: Phenomenon of Simpson's paradox in the association of *BTNL2:R281K* with SP.
