# Supplementary material for: Two Variants in the NOTCH4 and HLA-C Genes Contribute to Familial Clustering of Psoriasis
Source: Int J Genomics. 2020 Oct 19;2020:6907378. doi: 10.1155/2020/6907378 (PMC7593743; doi:10.1155/2020/6907378)
Supplement: Supplementary Materials — Table S1A: Detailed stepwise association results of NOTCH4:G534S and BTNL2:R281K with psoriasis risk. Table S1B: Phenomenon of Simpson's paradox in the association of BTNL2:R281K with SP. [file 6907378.f1.docx]

Table S1A. Detailed stepwise association results of *NOTCH4:G511S* and *BTNL2:R281K* with psoriasis risk.

| **variants** | **Analysis** | **Nominal association** | | | **Condition on  *HLA-C*06:02*** | | **Condition on  *HLA-C*** | | | **Condition on  *HLA-C, B*** | | |
| --- | --- | --- | --- | --- | --- | --- | --- | --- | --- | --- | --- | --- |
|  |  | **OR (95%CI)** | ***P*** | | **OR (95%CI)** | ***P*** | **OR (95%CI)** | | ***P*** | **OR (95%CI)** | ***P*** | |
| ***NOTCH4:G511S*** | **PFH vs control** | **5.72 (4.84, 6.76)** | **1.10E-92** | | **2.12 (1.75, 2.57)** | **1.38E-14** | **2.15 (1.77, 2.61)** | | **7.22E-15** | **2.10 (1.72, 2.55)** | **1.41E-13** | |
|  | **SP vs control** | **4.79 (4.21, 5.47)** | **1.23E-121** | | **1.69 (1.46, 1.97)** | **7.45E-12** | **1.71 (1.47, 1.99)** | | **2.84E-12** | 1.42 (1.19, 1.69) | 7.23E-05 | |
| ***BTNL2:R281K*** | **PFH vs control** | 0.68 (0.57, 0.82) | 4.29E-05 | | 1.08 (0.88, 1.32) | 0.47 | 1.06 (0.87, 1.31) | | 0.55 | 1.1 (0.89, 1.38) | 0.37 | |
|  | **SP vs control** | 0.93 (0.85, 1.03) | 0.19 | | 1.37 (1.21, 1.55) | 6.80E-07 | 1.35 (1.19, 1.53) | | 2.38E-06 | **1.45 (1.28, 1.65)** | **1.11E-08** | |
| Continued on next page …  Table S1A. Detailed stepwise association results of *NOTCH4:G511S* and *BTNL2:R281K* with psoriasis risk. | | | | | | | | | | | | |
| **variants** | **Analysis** | **Condition on  *HLA-C, B, DPB1*** | | | **Condition on  *HLA-C, B, DPB1, A*** | | **Condition on *HLA-C, B, DPB1, A, TAP2*** | | | **Condition on  *HLA-C, B, DPB1, A, TAP2, BTNL2*** | | |
|  |  | **OR (95%CI)** | | ***P*** | **OR (95%CI)** | ***P*** | **OR (95%CI)** | ***P*** | | **OR (95%CI)** | | ***P*** |
| ***NOTCH4:G511S*** | **PFH vs control** | **2.12 (1.74, 2.58)** | | **1.07E-13** | **2.11 (1.73, 2.57)** | **1.71E-13** | **2.04 (1.67, 2.49)** | **1.88E-12** | | **2.04 (1.68, 2.49)** | | **1.62E-12** |
|  | **SP vs control** | 1.45 (1.22, 1.72) | | 2.96E-05 | 1.49 (1.25, 1.77) | 7.63E-06 | 1.48 (1.25, 1.76) | 8.90E-06 | | 1.46 (1.22, 1.73) | | 2.50E-05 |
| ***BTNL2:R281K*** | **PFH vs control** | 1.07 (0.85, 1.34) | | 0.57 | 1.11 (0.90, 1.38) | 0.33 | 1.13 (0.91, 1.40) | 0.28 | | / | | / |
|  | **SP vs control** | **1.43(1.26, 1.62)** | | **5.30E-08** | **1.46(1.29, 1.66)** | **7.18E-09** | **1.49 (1.31, 1.70)** | **1.73E-09** | | / | | / |

Variants with *P* < 1.87 × 10^−6^ are marked in bold

Table S1B. Phenomenon of Simpson’s paradox in the association of *BTNL2:R281K* with SP

| **variant** | **Analysis** | **Freq_cases** | **Freq_controls** | **OR** | ***P*** |  |
| --- | --- | --- | --- | --- | --- | --- |
| *BTNL2:R281K* | SP vs control (all) | 0.059 | 0.063 | 0.93 | 0.19 |  |
|  |  |  |  |  |  |  |
|  | SP vs control (C0602-negative group) | 0.086 | 0.070 | 1.261 | 0.0112 |  |
|  | SP vs control (C0602-positive group) | 0.054 | 0.036 | 1.51 | 1.52E-05 |  |

Freq_cases = the allele frequency in patients, Freq_controls = the allele frequency in healthy controls
